# *In vitro* anti-biofilm activity of bacteriocin from a marine *Bacillus* sp. strain Sh10 against *Proteus mirabilis*

**Published:** 2020-02

**Authors:** Fatemeh Shayesteh, Asmat Ahmad, Gires Usup

**Affiliations:** 1 Department of Fisheries, Faculty of Marine Science and Technology, University of Hormozgan, Bandar Abbas, Iran; 2 Department of Bioscience and Biotechnology, Faculty of Science and Technology, University Kebangsaan Malaysia, Selangor, Malaysia; 3 Department of Environmental Science and Natural Resources, Faculty of Science and Technology, University Kebangsaan Malaysia, Selangor, Malaysia

**Keywords:** Marine bacteria, Bacteriocin, *Bacillus* sp., *Proteus mirabilis*, Anti-biofilm activity

## Abstract

**Background and Objectives:**

Biofilm formed by *Proteus mirabilis* strains is one of the most important medical problems especially in the case of device-related urinary tract infections. This study was conducted to evaluate the bacteriocin produced by a marine isolate of *Bacillus* sp. Sh10, for it's *in vitro* inhibitory activity against pre-formed biofilm and in interference with the biofilm-forming of two biofilm-producing bacteria (*P. mirabilis* UCa4 and *P. mirabilis* UCe1).

**Materials and Methods:**

Sensitivity of two biofilm-producing bacteria (*P. mirabilis* UCa4 and *P. mirabilis* UCe1) to bacteriocin, was investigated in planktonic and biofilm states by cell viability and crystal violet assay, respectively. Scanning electron microscopy (SEM) was also performed to determine the effect of bacteriocin on the morphology of the cells associated with biofilm.

**Results:**

It was found that bacteriocin possessed bactericidal activity to biofilm-forming isolates in the planktonic state. However, bacteriocin interferes with the formation of biofilms and disrupts established biofilms. Bacteriocin reduced biofilm formation in the isolates of *P. mirabilis* UCa4 and *P. mirabilis* UCe1 with SMIC_50_ of 32 and 128 μg/mL, desirable SMIC_50_ of bacteriocin for biofilm disruption were 128 and 256 μg/mL, respectively. The SEM results indicated that bacteriocin affected the cell morphology of biofilm-associated cells.

**Conclusion:**

The present findings indicated that bacteriocin from *Bacillus* sp. Sh10 has bactericidal properties against biofilm-forming isolates of *P. mirabilis* UCa4 and *P. mirabilis* UCe1 and has the ability to inhibit the formation of biofilm and disrupt established biofilm.

## INTRODUCTION

*Proteus mirabilis* is a member of the Enterobacteriaceae family and causes many clinical infections including those of the urinary tract, bloodstream, abdominal cavity and indwelling devices including scleral buckles, vascular access ports, urethral catheters, ureteral stents, and tracheoesophageal fistulas (1, 2). Among these clinical infections, *P. mirabilis* is the third most commonly isolated pathogen (after *Escherichia coli* and *Klebsiella pneumoniae*) of urinary tract infections (UTI) (3). An important adaptation of the bacteria *Proteus* spp., to cause infections in the urinary system, is the ability to form a biofilm (4). Microbial biofilms have been defined as sessile communities characterized by cells that are irreversibly attached to a substratum or interface or to each other, embedded in a matrix of extracellular polymeric substances that they produce, which exhibit an altered phenotype compared to their planktonic counterparts (5). This bacterial community is highly resistant to antimicrobials compared to their planktonic analogues. Thus, the treatment of biofilms with antibiotics or other biocides is usually ineffective at eradicating them (6, 7).

Based on several studies, *P. mirabilis* develops an increase resistant to some conventional antibiotics including ciprofloxacin (8–10) and fluoroquinolones (10, 11). As a result, novel therapeutic solutions other than the conventional antimicrobial therapies are an urgent need. Bacteriocins have attracted attention as potential substitutes for, or as additions to, currently used antimicrobial compounds, as such molecules are stable and potent against multidrug-resistant strains (12). Bacteriocins are inhibitory peptides or proteins produced by a group of bacteria. They elicit a bacteriocidal effect on different microbial groups closely related to the corresponding producer (13). These antimicrobial peptide compounds (AMPs) have been widely explored because of their high diversity, rapid mechanism of action, broad-spectrum activity, heat and pH stability, non-toxicity, and relatively cost-effective production (14, 15).

Recently, we reported that a bacteriocin producing isolate *Bacillus* sp. Sh10, which was isolated from the carpet clam *(Paphia textile)*, possessed a broad spectrum of inhibitory activity against different foodborne and human pathogens (16, 17). The exploitation of bacteriocins for microbial biofilm control is a relatively new research field. In this study, we investigated the antibacterial effects of bacteriocin produced by *Bacillus* sp. Sh10 against planktonic and biofilm cells of two biofilm-producing isolates (*P. mirabilis* UCa4 and *P. mirabilis* UCe1) from catheter-associated urinary tract which is an important cause of medical device-associated infections.

## MATERIALS AND METHODS

**Biofilm producing isolates.** Two biofilm-producing bacteria (*P. mirabilis* UCa4 and *P. mirabilis* UCe1) isolated from a urinary catheter used in surgeries at University Kebangsaan Malaysia Medical Center, were selected in this study. The selected isolates were examined for their ability to produce biofilm previously (18) and categorized as active biofilm former as described by Djordjevic et al. (19).

**Extraction of bacteriocin.** The bacteriocin-producing isolate *Bacillus* sp. Sh10 was isolated from the carpet clam *(Paphia textile)* using marine agar medium and was cultured in optimized medium containing inorganic salts (20), 1% glucose, 2% tryptone, and 2% sodium chloride (NaCl). The culture medium was adjusted to pH 8.0 and bacteria were incubated at 30 °C under aerobic condition (16). After 30 h, the cells were removed by centrifugation at 3000 × g for 30 min at 4 °C. The obtained supernatant was then passed through a membrane filter (0.22 μm) and was precipitated with ammonium sulfate at 80% of saturation for 24 h at 4 °C with gentle stirring. The precipitated protein was extracted by centrifugation at 3000 × g for 30 min. The obtained protein was dissolved in phosphate buffer (0.1 M, pH 7.0), dialyzed through a 2 kDa cut-off dialysis membrane (Sigma) against the same buffer at 4 °C for 24 h, and designed as ammonium sulfate fraction of bacteriocin (21).

**Bacteriocin susceptibility assay.** The antimicrobial activity of bacteriocin against biofilm-producing bacteria was determined using the spot-on-lawn method (22). In brief, 10 μL of the cell-free supernatant of Sh10 isolate obtained from the optimized medium was spotted onto the surface of Mueller Hinton agar overlaid with 1.5 × 10^8^ CFU/mL cells of the biofilm-producing organisms and then incubated at 37 °C. After 24 h of incubation, the zone of growth inhibition was observed.

**Determination of the minimum inhibitory concentration (MIC) and minimum bactericidal concentration (MBC).** The MICs and MBCs of bacteriocin for the planktonic cells of the selected biofilm-producing microorganisms were determined using the micro-dilution method and applying the procedures recommended by the Clinical and Laboratory Standards Institute (CLSI) (23). To prepare planktonic cells, three colonies of bacteria cultured overnight were inoculated on tryptone agar plates into 5 mL of tryptone soy broth, incubated at 37 °C until the mid-log phase was reached, and shaken at 100 rpm to avoid clump formation. The suspensions were then diluted to reach a bacterial density of approximately 1.5 × 10^8^ CFU mL^−1^. The diluted bacterial suspension (50 μL) was added to an equal volume of ammonium sulfate fraction of bacteriocin with different concentrations (0.5, 1, 2, 4, 8, 16, 32, 64, 128, 256, 512 and 1024 μg). The diluted suspensions were then incubated for 18–24 h at 37 °C. The MIC was the lowest concentration of bacteriocin that prevented visible turbidity after 18 or 24 h of incubation in tryptone soy broth. After MICs were determined, 10 μL of the solutions from the clear wells were plated onto Mueller Hinton agar and incubated at 37 °C overnight. The MBCs of bacteriocin was defined as the lowest concentrations of bacteriocin required to eradicate a particular bacterium.

**Effects of bacteriocin on the viability of the planktonic cells of biofilm-producing isolates.** Biofilm-producing isolates were grown to the midlog phase at 37 °C in tryptone soy broth medium and centrifuged at 3000 × g for 10 h. The cells were washed twice with 5 mmol L^−1^ sodium phosphate buffer (pH 7.0) and resuspended in the same medium; turbidity was adjusted to 0.5 McFarland. The ammonium sulfate fraction of bacteriocin was added to the cultures to reach the final concentration of 1 × MIC. The samples were subsequently incubated for 10 h. The inhibitory effect of bacteriocin on planktonic cell growth was determined as a change in OD_600_ and in cell viability at different time intervals. The number of viable cells was determined as CFU after 48 h of incubation for bacteria in tryptone soy agar plates. The cultures without bacteriocin were used as a control sample.

**Biofilm formation.** Biofilms were formed on 96-well commercially available pre-sterilized, flat-bottomed polystyrene microtiter plates. The biofilms were formed by adding 20 μL of an overnight culture of the biofilm-producing isolates containing 1.5 × 10^8^ CFU/mL to each well with 180 μL of sterile medium. After 48 h, the medium was aspirated, and the non-adherent cells were removed by thoroughly washing the biofilms thrice with 5 mmol L^−1^ sodium phosphate buffer (pH 7.0) without disrupting the biofilm on the wells. Biofilm formation was determined using crystal violet (24). In brief, the plates were dried for 1 h at 60 °C and stained with 2% crystal violet for 15 min. Excess stain was removed by rinsing the plates with tap water; crystal violet was re-dissolved in 95% ethanol, and OD_595_ was determined spectrometrically. Absorbance indicated the thickness of biofilm. Wells containing a sterile medium was designed as a blank sample.

**Effects of bacteriocin on biofilm formation.** Different dilutions of ammonium sulfate fraction of bacteriocin in sterile medium (0.5, 1, 2, 4, 8, 16, 32, 64, 128, 256, 512 and 1024 μg/mL) were used to examine whether bacteriocin could prevent the formation of biofilm and determine the lowest concentration of bacteriocin capable of preventing 50% of biofilm formation (SMIC_50_) in the selected isolates. The aliquots (180 μL) of each dilution were dispensed into the wells of the microtiter plate. The cell cultures (20 μL) were added to each well and then incubated for 48 h. SMIC_50_ of biofilm formation was compared with the results obtained in the bacteriocin-free control wells used in the crystal violet assay.

**Effects of bacteriocin on the established biofilm.** Biofilms were formed by adding the cell suspensions to the selected wells of the microtiter plate to determine whether bacteriocin can disrupt the preformed biofilm of the selected isolates. The biofilms were then incubated for 48 h. The biofilms were washed thoroughly thrice with 5 mmol L^−1^ sterile sodium phosphate buffer (pH 7.0). The aliquots (180 μL) of each dilution of ammonium sulfate fraction of bacteriocin (0.5, 1, 2, 4, 8, 16, 32, 64, 128, 256, 512 and 1024 μg/mL) were dispensed into the wells of the microtiter plate. The cell cultures (20 μL) were added to each well and further incubated for 24 h. A series of bacteriocin-free wells were used as a control sample. The MICs of sessile cells were determined at 50% disruption (SMIC_50_) of the preformed biofilm, and the results were compared with those obtained from the bacteriocin-free control wells in the crystal violet assay.

**SEM observation of biofilm treated with bacteriocin.** SEM was performed to determine the effects of bacteriocin on biofilm cells. The biofilms of the selected strains were grown as described previously, except that biofilm was formed on a urinary catheter. In brief, a sterile Foley catheter was cut into 1 cm pieces and then placed into a 96-well microtiter plate (one piece per well). After 48 h, the catheter pieces were transferred into new microtiter plates and then washed with sodium phosphate buffer thrice to remove non-adherent cells. Ammonium sulfate fraction bacteriocin was added to the samples to reach the final concentration of SMIC_50_ required to disrupt the biofilm in each sample. The plates were subsequently incubated for another 24 h. The control samples were incubated in a sterile medium only. After incubation was performed, the catheter samples were washed with sodium phosphate buffer thrice and placed in a fixation solution of 2% (v/v) glutaraldehyde in sodium phosphate buffer overnight at 4 °C. The samples were then dehydrated in a series of ethanol from 30% to 100% and then subjected to Critical Point Drying. The samples were sputtered with gold by using a polaron coater and viewed in a Philips XL 300 SEM.

**Statistical analysis.** All the essays in this study were carried out in triplicate. Experimental results were expressed as means ± standard deviation (SD) of three parallel measurements using Microsoft Excel software.

## RESULTS

**Determination of MIC and MBC for planktonic cells of biofilm-forming isolates.** The planktonic cells of the selected isolates were highly susceptible to bacteriocin. The MICs were 1 and 2 μg/mL for isolates *P. mirabilis* UCa4 and *P. mirabilis* UCe1, respectively. However, the MBCs were generally higher than the MIC for each isolate (Table 1).

**Table 1.** Determination of MIC, MBC and SMIC_50_ (μg/mL) of bacteriocin against biofilm-forming isolates

**Isolates**	**MIC**	**MBC**	**SMIC_50_**	

			**Established biofilm**	**Biofilm formation**
*P. mirabilis* UCa4	1	2	128	32
*P. mirabilis* UCe1	2	4	256	128

Minimum bacteriocin concentration that disrupts 50% of established biofilm biomass compared to control as determined by the crystal violet method.

**Fig. 1.**
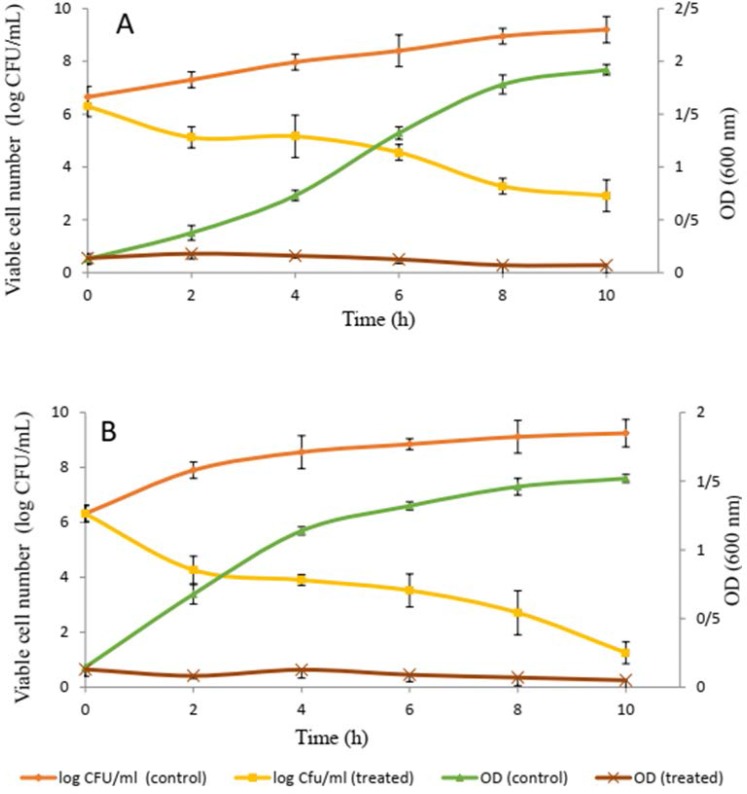
Time kill curve of biofilm-producing isolates at the planktonic form expose to ammonium sulfate precipitate fraction of bacteriocin at mid-logarithmic phase; A, *P. mirabilis* UCa4; B, *P. mirabilis* UCe1

**Effect of bacteriocin on the viability of planktonic cells of biofilm-forming isolates.** The addition of gel filtrate fraction of bacteriocin to a cell suspension of isolates caused a large decrease in the number of viable cells compared with the control of all samples over a period of 10 h, indicating that bacteriocin has a bactericidal effect on sensitive cells. However, the OD_600_ of bacteriocin-treated cells decreased after 10 h of incubation. This finding demonstrates that the antimicrobial substances induce cell lysis after cell death. By contrast, the OD_600_ of the untreated samples increased after 10 h of incubation (Fig. 1).

**Fig. 2.**
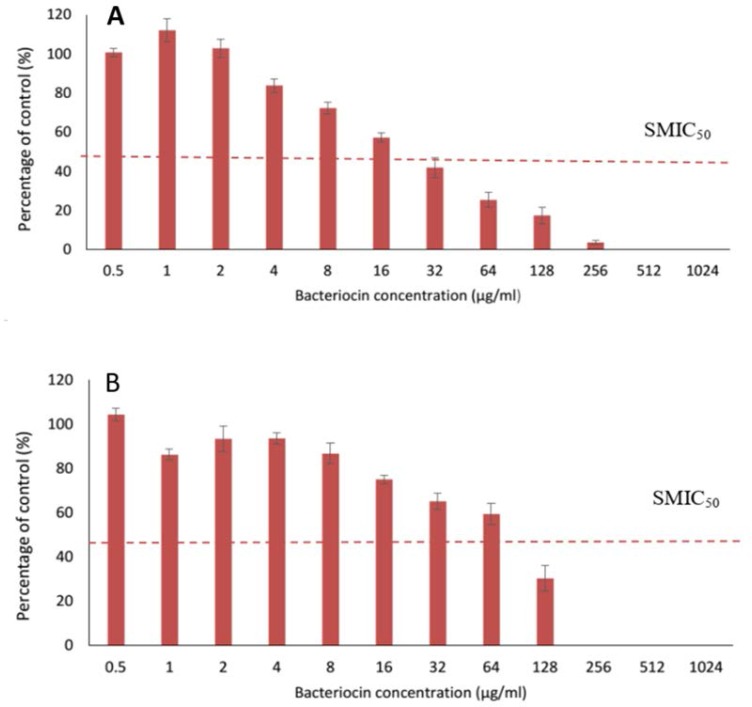
Effect of different concentration of bacteriocin on the biofilm formation of selected isolates; A, *P. mirabilis* UCa4; B, *P. mirabilis* UCe1. Dotted line indicates a range of probable SMIC_50_ values.

**Effect of bacteriocin on biofilm formation.** The inhibition of biofilm formation was dependent on the concentration of bacteriocin. Bacteriocin reduced biofilm formation in the isolates *P. mirabilis* UCa4 and *P. mirabilis* UCe1 with SMIC_50_ of 32 and 128 μg/mL respectively (Table 1). In addition, the bacteriocin concentrations for complete biofilm-forming prevention for these isolates were 512 and 256 μg/mL, respectively (Fig. 2).

**Fig. 3.**
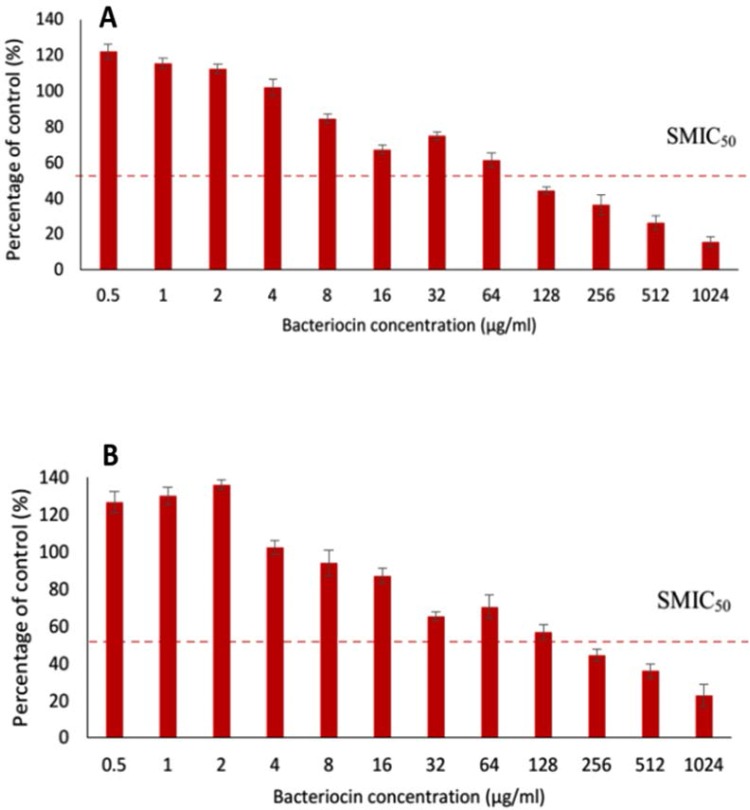
Effect of different concentration of bacteriocin on the established biofilm of selected isolates; A, *P. mirabilis* UCa4; B, *P. mirabilis* UCe1. Dotted line indicates a range of probable SMIC_50_ values.

**Effect of bacteriocin on established biofilm.** The desirable SMIC_50_ of bacteriocin for biofilm disruption for the isolates *P. mirabilis* UCa4 and *P. mirabilis* UCe1 were 128 and 256 μg/mL respectively (Table 1). However, the concentrations of bacteriocin applied in this assay were insufficient for complete eradication of the established biofilm (Fig. 3). These findings demonstrated that a higher concentration of bacteriocin is required to disrupt established biofilm than to prevent biofilm formation.

**Fig. 4.**
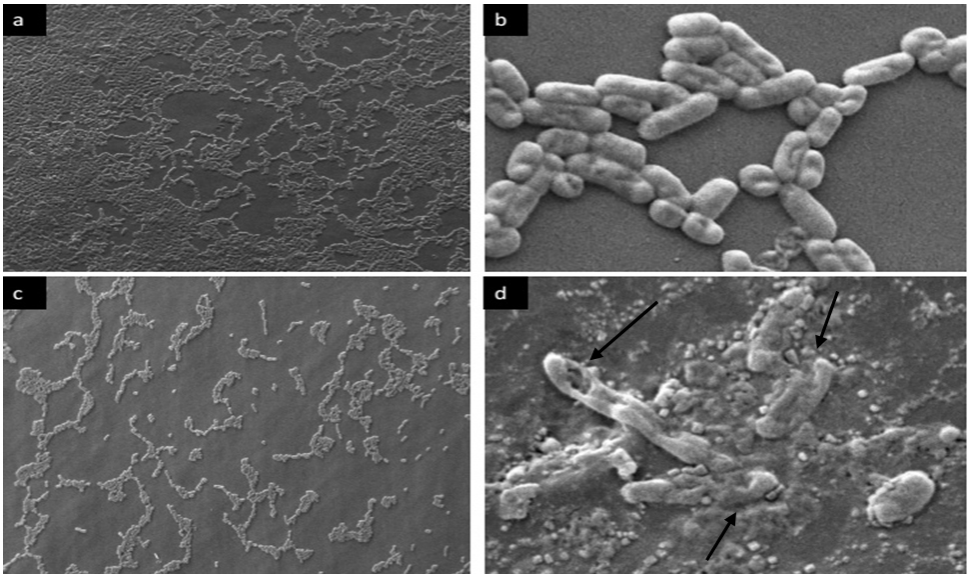
SEM observation of control and bacteriocin-treated *P. mirabilis* UCa4 biofilm formed on a urine catheter. (a) and (b) untreated control biofilms; (c) and (d) established biofilm exposed to bacteriocin (SMIC_50_) for 24 h. Magnification 10.00 KX (a, c) and 1.00 KX (b, d). Black arrows indicating abnormal treated cells.

**Fig. 5.**
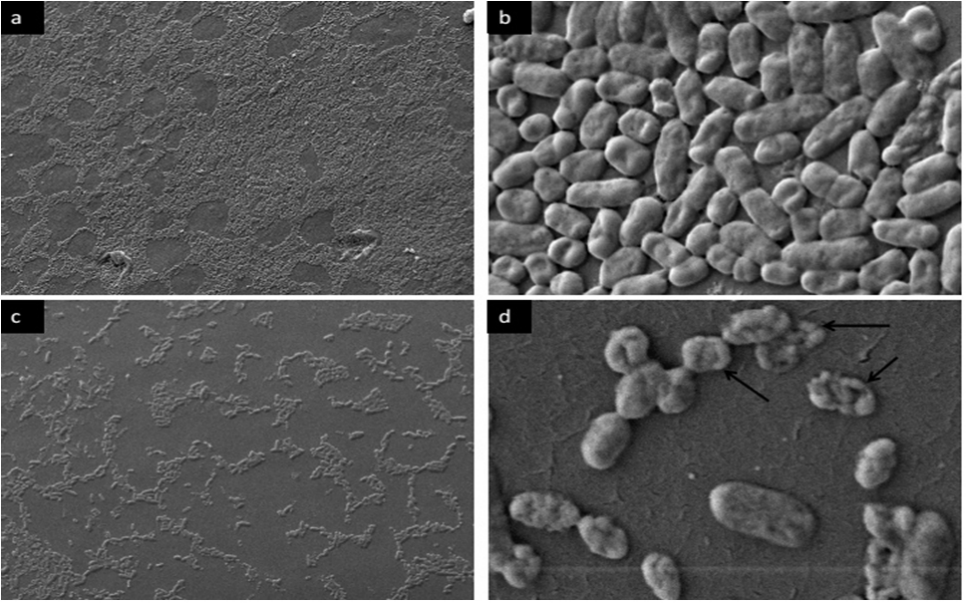
SEM observation of control and bacteriocin-treated *P. mirabilis* UCe1 biofilm formed on a urine catheter. (a) and (b) untreated control biofilms; (c) and (d) established biofilm exposed to bacteriocin (SMIC_50_) for 24 h. Magnification 1.00 KX (a, c) and 10.00 KX (b, d). Black arrows indicating abnormal treated cells.

**SEM analysis of preformed biofilm cells after-exposure to bacteriocin.** SEM was employed to evaluate the effect of bacteriocin on the morphology of the cells associated with preformed biofilm. The untreated cells were intact (regular rod) and showed smooth surfaces, as shown in Figs. 4 and 5, whereas cells treated with bacteriocin showed morphological changes. After exposure of bacteriocin to preformed biofilm with required SMIC_50_ for biofilm disruption, biofilm cells showed rough and well-defined wrinkles of the cell wall. Unlike smooth surface and intact untreated cells, dissociation of some part of the cell was also observed. These observations clearly suggested the damaging effects of bacteriocin on the surface layers, thereby confirming the bactericidal effect of bacteriocin on the cells associated with biofilms.

## DISCUSSION

Quantitative measurements of biofilm by crystal violet along with CFU measurement and SEM observations indicated the ability of bacteriocin produced by *Bacillus* sp. Sh10, to reduce the biomass of biofilm (Figs. 2–4 and 5). These findings suggesting that bacteriocin may exert an inhibitory effect on extracellular matrix accumulation/production thereby reducing biofilm-associated bacterial cells. Based on previous reports (18, 19), it is assumed that biofilms can be indirectly quantified by crystal violet assay. Crystal violet does not selectively bind to a specific biofilm component but binds to all substances in the biofilm. Crystal violet assay, therefore, measures total biomass, including both biofilm attached cells and exopolysaccharide (EPS). Moreover, the addition of gel filtrate fraction of bacteriocin to a planktonic cell suspension of biofilm-producing isolates, caused a large decrease in the number of viable cells compared with the control of all samples over a period of 10 h, indicating that bacteriocin has a bactericidal effect on sensitive cells.

This work demonstrated significant differences in bacteriocin susceptibility between planktonic and biofilm populations of the same organisms. A similar result was reported by Olszewska et al. who compared the MIC of planktonic and biofilm cells of *Pseudomonas aeruginosa* ATCC 27853 to two disinfectants, chlorine-based and quaternary ammonium compound-based (25). They revealed that biofilm cells were more tolerant to the chlorine-based sanitiser than planktonic counterparts. Chylkova et al. compared the MIC of planktonic and biofilm cells of five *Salmonella* sp. isolates against common antimicrobial disinfectants (i.e., peroxyacetic acid, acidified hypochlorite, and cetylpyridinium chloride) and found that the isolates were sensitive to these antimicrobial agents in their planktonic form but highly resistant to the same disinfectants in their biofilm forms (26).

Cells existing in biofilm form are up to 1000 times more resistant to antimicrobial agents (27). Biofilm resistance can be partly explained by the following hypotheses. In the first hypothesis, EPS secreted by biofilm cells acts as a physical/chemical barrier to prevent penetration by many antibiotics (31). EPS is negatively charged and functions as an ion-exchange resin capable of binding the antibiotic molecules attempting to reach the embedded biofilm cells. The second hypothesis is based on changes that occurin the biofilm micro-environment. According to this hypothesis, some biofilm cells survive by falling into a state of slow growth because of the lack of nutrients or the accumulation of harmful metabolites (28). According to the third hypothesis, biofilms contain dormant persister cells that are largely responsible for multidrug tolerance (29). Persisters can survive despite antibiotic concentrations well above the MIC.

The anti-biofilm mechanisms of action of AMPs are still poorly investigated. For instance, some peptides can interfere with the early events of biofilm formation by preventing bacterial cell adhesion to the substrate or to other cells, or by killing cells before they stably become part of the biofilm architecture. Others may act on established biofilms by killing or detaching mature biofilm cells. Overhage et al. reported that the human cathelicidin antimicrobial peptide LL-37 prevents *P. aeruginosa* biofilm formation (30). This antimicrobial peptide shows activity against pre-formed (2 days old) *P. aeruginosa* biofilms and reduces 60% of biofilm thickness. According to Park et al. some bacteriocins can be transferred in biofilm EPS through pores formed in the lipid component of the EPS (31). This phenomenon enables the bacteriocin to penetrate the EPS and eradicate EPS-embedded bacteria. Antimicrobial peptides have a high potential to also act on slow-growing, non-growing, or persister cells because they permeabilize and/or to form pores in the cytoplasmic membrane (32). For instance, a synthetic cationicpeptide, (RW)4–NH2, can kill more than 99% of *E. coli* HM22 persister cells in planktonic culture. This synthetic peptide can also reduce the number of persister cells inmature biofilms up to 98% (33).

High and low magnifications were used in SEM observation to distinguish the possible morphological and density changes between treated and untreated biofilm-associated cells. Low-magnification SEM observation showed a gradual decrease in cell density after bacteriocin treatment compared with untreated established biofilm, suggesting that bacteriocin can disassociate biofilm cells from the urine catheter by degrading biofilm matrix materials. High-magnification SEM images revealed the damaged cell morphology of the tested pathogens, showing large surface collapse, abnormal cell breakage, wrinkled cell walls, and completely lysed or dead cell formations. These results show that bacteriocin treatment altered the cell outer membrane. The cell membrane is the main barrier limiting the distribution and entry of antimicrobial compounds (34). In addition to antimicrobial activities, bacteriocins interact with bacterial cell membranes and create ion-permeable channels, leading to increased cytoplasmic membrane permeability, osmotic balance disruption and, hence, bacterial cell death (32). In a result, the bacteriocin produced by the marine isolate *Bacillus* sp. Sh10 could be a potential anti-biofilm therapeutic candidate. Unlike most antibiotics, bacteriocin does not target cellular components such as nucleic acids and proteins, leading to the resistance development of targeted bacteria against such antibiotics.

In conclusion, the findings indicate that bacteriocin from *Bacillus* sp. Sh10 can inhibit *P. mirabilis* biofilms. The significant antibacterial activity of bacteriocin suggests that this could serve as a source of compounds which have therapeutic potential for the treatment of *P. mirabilis* infections. Further *in vivo* evaluations are required to determine whether these findings can be exploited in treating biofilm-associated *Proteus* infections.
